# Dose–response relationship between genetically proxied average blood glucose levels and incident coronary heart disease in individuals without diabetes mellitus

**DOI:** 10.1007/s00125-020-05377-0

**Published:** 2021-01-26

**Authors:** Stephen Burgess, Rainer Malik, Bowen Liu, Amy M. Mason, Marios K. Georgakis, Martin Dichgans, Dipender Gill

**Affiliations:** 1 Medical Research Council Biostatistics Unit, Cambridge Institute of Public Health, Cambridge, UK; 2 Department of Public Health and Primary Care, University of Cambridge, Cambridge, UK; 3 Institute for Stroke and Dementia Research, University Hospital of Ludwig-Maximilians-University, Munich, Germany; 4 Munich Cluster for Systems Neurology, Munich, Germany; 5 German Centre for Neurodegenerative Diseases, Munich, Germany; 6 Clinical Pharmacology and Therapeutics Section, Institute of Medical and Biomedical Education and Institute for Infection and Immunity, St George’s, University of London, London, UK; 7 Clinical Pharmacology Group, Pharmacy and Medicines Directorate, St George’s University Hospitals NHS Foundation Trust, London, UK; 8 Novo Nordisk Research Centre Oxford, Old Road Campus, Oxford, UK; 9 Department of Epidemiology and Biostatistics, School of Public Health, Imperial College London, London, UK

**Keywords:** Average blood glucose levels, CHD, Mendelian randomisation

## Abstract

**Aims/hypothesis:**

Our aim was to investigate the relationship between average blood glucose levels and incident CHD in individuals without diabetes mellitus.

**Methods:**

To investigate average blood glucose levels, we studied HbA_1c_ as predicted by 40 variants previously shown to be associated with both type 2 diabetes and HbA_1c_. Linear and non-linear Mendelian randomisation analyses were performed to investigate associations with incident CHD risk in 324,830 European ancestry individuals from the UK Biobank without diabetes mellitus.

**Results:**

Every one mmol/mol increase in genetically proxied HbA_1c_ was associated with an 11% higher CHD risk (HR 1.11, 95% CI 1.05, 1.18). The dose–response curve increased at all levels of HbA_1c_, and there was no evidence favouring a non-linear relationship over a linear one.

**Conclusions/interpretations:**

In individuals without diabetes mellitus, lowering average blood glucose levels may reduce CHD risk in a dose-dependent way.

**Graphical abstract:**

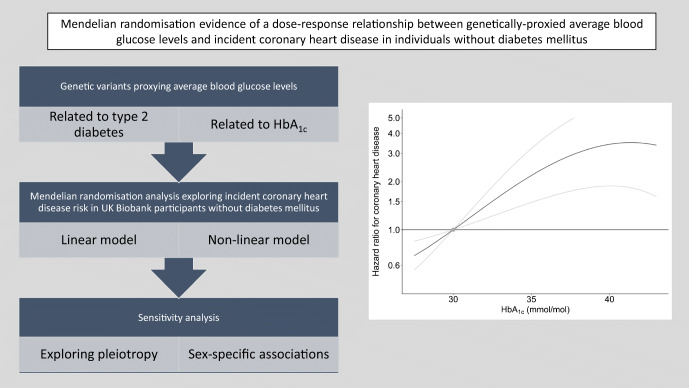

**Supplementary Information:**

The online version of this article (10.1007/s00125-020-05377-0) contains peer-reviewed but unedited supplementary material.

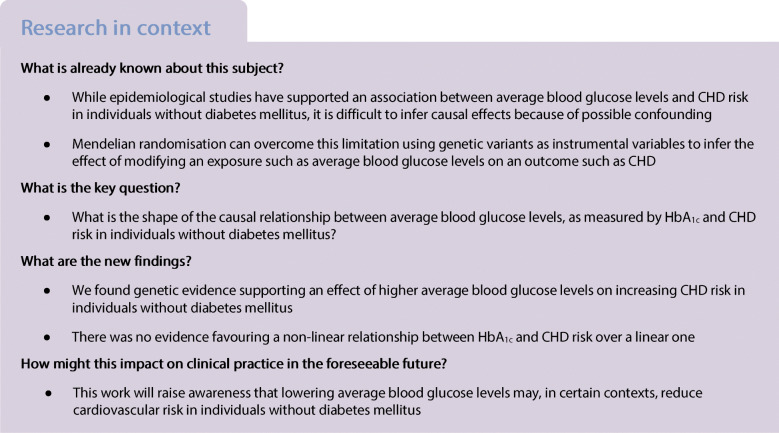



## Introduction

CHD is a leading cause of mortality, accounting for approximately 9 million deaths globally in 2015 alone [1]. Epidemiological studies have supported an association between average blood glucose levels and CHD risk, even in individuals without diabetes mellitus [2]. However, it is difficult to infer causal effects from observational studies because of the possibility that any identified associations may be attributable to confounding. Mendelian randomisation can overcome these limitations by using genetic variants as instrumental variables to infer the effect of modifying an exposure such as average blood glucose levels on an outcome such as CHD. The aim of this study was to perform linear and non-linear Mendelian randomisation analyses to investigate the shape of the causal relationship between average blood glucose levels (measured by HbA_1c_) and CHD risk in individuals without diabetes mellitus. HbA_1c_ was considered preferable to blood glucose levels because it represents the average blood glucose level over approximately 120 days, so is less susceptible to variation related to the time of recording.

## Methods

Analyses were performed in unrelated participants of European ancestry from the UK Biobank, a population-based cohort study of middle-aged UK residents. Individuals having possible diabetes mellitus (defined based on self-reporting, hospital episode statistics and medications) or baseline HbA_1c_ > 47.5 mmol/mol (6.5%) were excluded. HbA_1c_ was measured in packed red blood cells using the Bio-Rad Variant II Turbo analyser. International Classification of Diseases 9th Revision (ICD-9) codes 410-414, and ICD-10 codes I20-I25 were used to identify incident CHD cases. Full details are provided in the electronic supplementary material (ESM).

### Candidate instrumental variables

We selected 40 uncorrelated (r^2^ < 0.001) single-nucleotide polymorphisms as instrumental variables for average blood glucose levels based on their association with type 2 diabetes (*p* < 5 × 10^−8^) in a genome-wide association study of 228,499 cases and 1,178,783 controls (79% European ancestry) that included UK Biobank participants [3], and their association with HbA_1c_ (*p* < 0.001 and concordant direction of association) in an independent study of 100,880 European ancestry participants (no overlap with the UK Biobank) that were free of diabetes mellitus (as defined by physician diagnosis, medications, or fasting glucose ≥7 mmol/l) [4] (ESM Table 1). We created a weighted allele score for each participant by multiplying each type 2 diabetes risk-increasing allele dosage with the variant’s association with HbA_1c_, and summing across all 40 variants. Selecting variants associated with type 2 diabetes and weighting by their association with HbA_1c_ helps ensure that the weighted allele score is reflective of average blood glucose levels, rather than solely type 2 diabetes risk or HbA_1c_.

### Statistical analysis

Mendelian randomisation analyses were performed to investigate the association between genetically proxied average blood glucose levels (measured as HbA_1c_) and incident CHD. Analyses were performed by modelling a linear relationship between genetically proxied average blood glucose levels and incident CHD (‘linear Mendelian randomisation’) [5], and also using the fractional polynomial method to test for a non-linear relationship between genetically proxied average blood glucose levels and incident CHD (‘non-linear Mendelian randomisation’) [6]. We further assessed associations of the allele score with lipid fractions and other glycaemic traits, and performed multivariable non-linear Mendelian randomisation for traits associated with the allele score that may represent alternative causal risk factors. As a further sensitivity analysis, we also performed Mendelian randomisation analysis that excluded variants associated with the alternative causal risk factor at *p* < 0.01. Full details are provided in the ESM. All statistical analysis was performed using R (version 3.6.2) and only publicly available data from studies that had obtained relevant ethical approval and participant consent were used.

## Results

Baseline characteristics for the 324,830 participants included in the analyses are provided in ESM Table 2. There were 6006 incident CHD events. The allele score explained 1.8% of the variance in HbA_1c_, corresponding to an F-statistic of 144.5 and a low risk of substantial weak instrument bias. For UK Biobank participants, associations of the variants incorporated in the allele score with HbA_1c_ were generally of greater magnitude in men compared with women (ESM Fig. 1).

### Linear Mendelian randomisation

Linear Mendelian randomisation analyses identified a positive association between higher genetically proxied average blood glucose levels and incident CHD risk when considering men and women together (ESM Fig. 2). For a one mmol/mol increase in HbA_1c_, the HR for incident CHD was 1.11 (95% CI 1.05, 1.18; *p* = 2 × 10^−4^). In sex-stratified analyses, the association was stronger in men (HR 1.12, 95% CI 1.05, 1.19; *p* = 4 × 10^−4^) than in women (HR 1.08, 95% CI 0.96, 1.20; *p* = 0.20) (ESM Table 3). Similar point estimates were obtained in sensitivity analyses using alternative Mendelian randomisation methods (ESM Table 4).

### Non-linear Mendelian randomisation

**Fig. 1:**
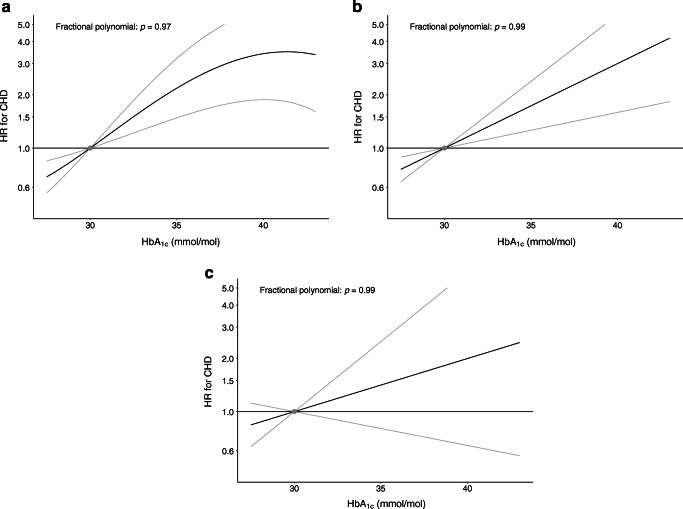
Non-linear Mendelian randomisation investigating the relationship between genetically proxied average blood glucose levels (measured by HbA_1c_) and risk of incident CHD in individuals without diabetes mellitus: (**a**) men and women combined; (**b**) men only; and (**c**) women only. The *x*-axis depicts HbA_1c_ levels in mmol/mol. The *y*-axis depicts the hazard ratio for coronary heart disease (HR for CHD) with respect to the reference, plotted on a log scale. Reference is set to an HbA_1c_ of 30 mmol/mol (4.9%). The grey lines represent the 95% CIs. The fractional polynomial test is a goodness-of-fit test that assesses whether any improvement of fit when using a non-linear function to model the association, compared with a linear function, is greater than would be expected due to chance (a significant *p* value indicates that a non-linear model is preferred to a linear model)

In non-linear Mendelian randomisation, we observed no statistical evidence favouring a non-linear relationship between genetically proxied HbA_1c_ and incident CHD over a linear one in any of the analyses (Fig. 1). Subgroup analyses presenting Mendelian randomisation estimates in quintiles of the population based on HbA_1c_ levels (corrected for genetic predisposition) are presented in ESM Table 3.

We assessed genetic associations of the allele score and its individual variants with other glycaemic traits and lipid fractions (ESM Table 5 and ESM Fig. 3). The allele score was associated with 2-h glucose (*p* < 0.001) and fasting glucose (*p* < 0.001), but not fasting insulin (*p* = 0.22). The allele score was also associated with LDL-cholesterol (LDL-cholesterol, *p* = 0.03), but not HDL-cholesterol (*p* = 0.86) or triacylglycerols (*p* = 0.99). Although multivariable non-linear Mendelian randomisation adjusting for genetically proxied LDL-cholesterol showed some attenuation in the coefficient for genetically proxied HbA_1c_, an association persisted (HR 1.07, 95% CI 1.01, 1.14; *p* = 0.018), and the best-fitting fractional polynomial was the linear model (ESM Fig. 4). Similar results were also obtained after excluding the five variants that associated with LDL-cholesterol at *p* < 0.01 (rs1260326, rs10184004, rs11708067, rs505922 and rs174541): HR 1.10 (95% CI 1.03, 1.17; *p* = 0.003) (ESM Fig. 5).

## Discussion

In this Mendelian randomisation study, we found genetic evidence supporting an effect of higher average blood glucose levels on increasing CHD risk in individuals without diabetes mellitus. We did not find evidence favouring a non-linear relationship between HbA_1c_ and CHD risk over a linear one. Our findings provide evidence that lowering average blood glucose levels in individuals without diabetes mellitus can reduce cardiovascular risk in a dose-dependent way.

While pre-diabetes is known to increase cardiovascular risk [7], our work goes further to support a continuous effect of average blood glucose levels that are within the ‘physiologically normal’ HbA_1c_ range. Our findings build on existing epidemiological research supporting an association between average blood glucose levels and CVD risk in individuals without diabetes mellitus [2]. A previous Mendelian randomisation study similarly showed a positive association between genetically proxied HbA_1c_ and CHD risk, although with wider CIs than in our current work, likely because that work used fewer genetic variants as instrumental variables [8]. Clinical trials have found that intensive lowering of HbA_1c_ levels in high-risk patients with type 2 diabetes does not confer a beneficial effect on cardiovascular risk [9, 10]. This discrepancy may be related to the particular pharmacological treatments used to lower HbA_1c_ levels, including adverse effects such as weight gain, hypoglycaemia and rapid fluctuations in glucose levels [9, 10]. Pharmacological agents for blood glucose lowering that are not associated with weight gain or hypoglycaemia are available [11]. Clinical trials are warranted to explore whether particular blood glucose lowering strategies can be used to reduce cardiovascular risk in patients without diabetes mellitus.

A strength of our study is that the employed Mendelian randomisation approach is robust to confounding from environmental factors. The weights for the variants used in Mendelian randomisation analysis were derived from their associations with HbA_1c_ in a dataset that did not include the UK Biobank [4]. Furthermore, analysis was restricted to UK Biobank participants free of type 2 diabetes, thus avoiding any influence of winner’s curse bias. We further performed sensitivity analyses that accounted for potential genetic confounding through LDL-C, which also identified associations of genetically proxied HbA_1c_ levels with incident CHD risk. However, our study also has limitations. The analyses were restricted to UK Biobank participants of European ancestry, and may not apply to other populations. Furthermore, the genetic variants employed as instrumental variables only explained 1.8% of the variance in HbA_1c_, thus limiting the statistical power of the analyses and the precision of the results. A reason why the Mendelian randomisation estimates were less precise in women compared with men may be that the genetic variants had weaker associations with HbA_1c_ in women. Finally, the genetic variants employed as instruments in this analysis proxy lifelong average blood glucose control, and therefore cannot be used to inform on the quantitative effects of discrete clinical interventions that lower blood glucose levels in the short term.

In summary, this Mendelian randomisation analysis provides genetic evidence supporting an effect of average blood glucose levels on CHD risk in individuals without diabetes mellitus. Further work is required to investigate whether strategies that lower blood glucose levels can reduce cardiovascular risk in individuals without diabetes mellitus.

## Supplementary Information

ESM (PDF 980 kb)

## Data Availability

This research was performed using UK Biobank data (application 29202), which is available on request (https://www.ukbiobank.ac.uk/register-apply). The genetic variants used as instrumental variables are detailed in ESM Table 1.
