# Supplementary material for: Dose–response relationship between genetically proxied average blood glucose levels and incident coronary heart disease in individuals without diabetes mellitus
Source: Diabetologia. 2021 Jan 26;64(4):845–9. doi: 10.1007/s00125-020-05377-0 (PMC7940279; doi:10.1007/s00125-020-05377-0)

Electronic supplementary material (ESM)

Dose-response relationship between genetically-proxied average blood glucose levels and incident coronary heart disease in individuals without diabetes mellitus

Stephen Burgess[1,2], Rainer Malik[3], Bowen Liu[2], Amy M. Mason[2], Marios K. Georgakis[3], Martin Dichgans[3-5], Dipender Gill[6-9*]

[1]Medical Research Council Biostatistics Unit, Cambridge Institute of Public Health, Cambridge, United Kingdom.

[2]Department of Public Health and Primary Care, University of Cambridge, Cambridge, United Kingdom.

[3]Institute for Stroke and Dementia Research (ISD), University Hospital of Ludwig-Maximilians-University (LMU), Munich, Germany.

[4]Munich Cluster for Systems Neurology (SyNergy), Munich, Germany.

[5]German Centre for Neurodegenerative Diseases (DZNE), Munich, Germany.

[6]Clinical Pharmacology and Therapeutics Section, Institute of Medical and Biomedical Education and Institute for Infection and Immunity, St George's, University of London, London, United Kingdom.

[7]Clinical Pharmacology Group, Pharmacy and Medicines Directorate, St George's University Hospitals NHS Foundation Trust, London, United Kingdom.

[8]Novo Nordisk Research Centre Oxford, Old Road Campus, Oxford, United Kingdom.

[9]Department of Epidemiology and Biostatistics, School of Public Health, Imperial College London, London, United Kingdom.

Contents

ESM Methods. 3
ESM Table 1. 14
ESM Table 2. 16
ESM Table 3. 17
ESM Table 4. 18
ESM Table 5. 19
ESM Figure 1. 20
ESM Figure 2. 21
ESM Figure 3. 22
ESM Figure 4. 23
ESM Figure 5. 24

ESM Methods

UK Biobank

The UK Biobank is made up of approximately 500,000 participants (of which 94% are of self-reported European-ancestry), aged 40 to 69 at recruitment between 2006 and 2010 across 22 assessment centres in the UK. Ethical approval for the UK Biobank study was obtained from the North West Multicenter Research Ethics Committee. All participants provided written informed consent. In this study, UK Biobank data was accessed through application 29202 and follow up was performed to 31 March 2020 or the date of death. Participant information was available for genotype, clinical measurements, biological assays, and self-reported health behaviours, with further linkage to electronic health records (1). To derive our initial analytic sample, we excluded participants having non-European ancestry (self-report or judged by genetics), low call rate or excess heterozygosity (>3 standard deviations from the mean) as described previously (2). We included only one of each set of related participants (third-degree relatives or closer). We also excluded participants without a valid HbA_{1c} measurement.

HbA_{1c} was measured in packed red blood cells using the Bio-Rad Variant II Turbo analyser (Bio-Rad Laboratories, Inc), which employs a High Performance Liquid Chromatography method. Results are expressed in mmol/mol units, with an analytical range of 15-184mmol/mol.

Our analyses only included participants who were judged as unlikely to have any type of diabetes mellitus. Possible diabetes was identified based on self-reported information, hospital episode statistics, and information on prescription medication as previously described (3). Only those judged as diabetes 'unlikely' were included in the analysis. Additionally, we excluded from analysis all those with residual HbA_{1c} (defined below) above

47.5 mmol/mol (6.5%), the threshold defined by the American Diabetes Association as a diagnostic criterion for diabetes (4).

International Classification of Diseases 9th Revision (ICD-9) codes 410-414, and ICD-10 codes I20-I25 were used to identify incident coronary heart disease cases.

Linear Mendelian randomization

The ratio of coefficients method was used to perform Mendelian randomization analyses that assumed a linear relationship between genetically-proxied average blood glucose levels and risk of incident coronary heart disease (CHD) (5). This represents the association of the average blood glucose level allele score with CHD divided by the association of the allele score with HbA_{1c} (6). We used linear regression to estimate the association of the allele score with HbA_{1c}, incorporating age, sex, principal components 1-10 of genetic ancestry, genotyping chip and assessment centre as covariates.

We calculated the proportion of variance in HbA_{1c} explained by the allele score and its F-statistic to estimate instrument strength (7). We used Cox proportional hazard regression to estimate the association of the allele score with CHD risk, incorporating sex, principal components 1-10 of genetic ancestry, genotyping chip and assessment centre as covariates. Age was used as the time variable in the time-to-event analyses. In sensitivity analyses, each variant in the allele score was considered as a separate instrumental variable using Mendelian randomization methods that differ in their requisite assumptions on the inclusion of pleiotropic variants: fixed-effects inverse-variance weighted, random-effects inverse-variance weighted, Egger, weighted median, contamination-mixture and PRESSO Mendelian randomization (8). An intercept term in the Egger method that differs from zero can be used

to provide evidence of directional pleiotropy (9). Statistics measuring heterogeneity in the Mendelian randomization estimates generated by different variants were further calculated to measure potential pleiotropy (10).

Non-linear Mendelian randomization

The fractional polynomial method was used to investigate for a non-linear relationship between genetically-proxied average blood glucose levels and risk of incident CHD (11-13). In this approach, we stratified the population into trigintiles (30 equal groups) based on residual HbA_{1c}, which is defined as a participant's HbA_{1c} minus the genetic contribution to HbA_{1c} from the average blood glucose level allele score. Thus, we aimed to compare individuals in the population who would have a similar average blood glucose levels (in the same trigintile stratum) if they also had the same genetic predisposition. Stratifying on HbA_{1c} itself would introduce collider bias and potentially distort estimates, as average blood glucose levels may be on the causal pathway from the genetic variants to CHD (13; 14). For each trigintile of the population, a linear Mendelian randomization estimate for the association of genetically-proxied HbA_{1c} with CHD was calculated using the ratio of coefficients method, as detailed above (6). A meta-regression of the linear Mendelian randomization estimates obtained for each trigintile against the mean HbA_{1c} in that centile was then performed using a flexible semiparametric framework (11; 13). We used a fractional polynomial test to investigate whether a non-linear model fit this meta-regression better than a linear model (11-13). A significant p value for this test is evidence against the null hypothesis that the linear model fits the data as well as the best-fitting fractional polynomial model. Hence a significant p value suggests that a non-linear model fits the data better than a linear model. Pre-specified

subgroup analyses considering males and females separately were also performed to investigate potential sex-specific effects.

Multivariable Mendelian randomization

Associations of the allele score were assessed using two-sample Mendelian randomization implemented by the inverse-variance weighted method with a random-effects model. Genetic associations with two-hour (post-load) glucose, fasting glucose, and fasting insulin were obtained from the MAGIC consortium (15; 16). Genetic associations with low-density lipoprotein (LDL) cholesterol, high-density lipoprotein (HDL) cholesterol, and triglycerides were obtained from the Global Lipids Genetics Consortium (GLGC) (17). Multivariable Mendelian randomization was performed by first creating an allele score for genetically-proxied LDL-cholesterol using genetic associations with LDL-cholesterol from the GLGC as weights. We then adjusted for genetically-proxied LDL-cholesterol in the calculation of the stratum-specific estimates, before combining in the non-linear model as described above.

Exclusion of variants associated with LDL-cholesterol

As a further sensitivity analysis, we performed Mendelian randomization analysis that excluded variants associated with LDL-cholesterol at $p<0.01$.

References

1. Bycroft C, Freeman C, Petkova D, Band G, Elliott LT, Sharp K, Motyer A, Vukcevic D, Delaneau O, O'Connell J, Cortes A, Welsh S, Young A, Effingham M, McVean G, Leslie S,

Allen N, Donnelly P, Marchini J. The UK Biobank resource with deep phenotyping and genomic data. Nature 2018;562:203-209

2. Astle WJ, Elding H, Jiang T, Allen D, Ruklisa D, Mann AL, Mead D, Bouman H, Riveros-Mckay F, Kostadima MA, Lambourne JJ, Sivapalaratnam S, Downes K, Kundu K, Bomba L, Berentsen K, Bradley JR, Daugherty LC, Delaneau O, Freson K, Garner SF, Grassi L, Guerrero J, Haimel M, Janssen-Megens EM, Kaan A, Kamat M, Kim B, Mandoli A, Marchini J, Martens JHA, Meacham S, Megy K, O'Connell J, Petersen R, Sharifi N, Sheard SM, Staley JR, Tuna S, van der Ent M, Walter K, Wang SY, Wheeler E, Wilder SP, Iotchkova V, Moore C, Sambrook J, Stunnenberg HG, Di Angelantonio E, Kaptoge S, Kuijpers TW, Carrillo-de-Santa-Pau E, Juan D, Rico D, Valencia A, Chen L, Ge B, Vasquez L, Kwan T, Garrido-Martin D, Watt S, Yang Y, Guigo R, Beck S, Paul DS, Pastinen T, Bujold D, Bourque G, Frontini M, Danesh J, Roberts DJ, Ouwehand WH, Butterworth AS, Soranzo N. The Allelic Landscape of Human Blood Cell Trait Variation and Links to Common Complex Disease. Cell 2016;167:1415-1429 e1419

3. Eastwood SV, Mathur R, Atkinson M, Brophy S, Sudlow C, Flaig R, de Lusignan S, Allen N, Chaturvedi N. Algorithms for the Capture and Adjudication of Prevalent and Incident Diabetes in UK Biobank. PLoS One 2016;11:e0162388

4. American Diabetes Association. 12. Older Adults: Standards of Medical Care in Diabetes-2020. Diabetes Care 2020;43:S152-S162

5. Burgess S, Small DS, Thompson SG. A review of instrumental variable estimators for Mendelian randomization. Stat Methods Med Res 2017;26:2333-2355

6. Burgess S, Thompson SG. Use of allele scores as instrumental variables for Mendelian randomization. Int J Epidemiol 2013;42:1134-1144

7. Palmer TM, Lawlor DA, Harbord RM, Sheehan NA, Tobias JH, Timpson NJ, Davey Smith G, Sterne JA. Using multiple genetic variants as instrumental variables for modifiable risk factors. Stat Methods Med Res 2012;21:223-242

8. Slob EAW, Burgess S. A comparison of robust Mendelian randomization methods using summary data. Genet Epidemiol 2020;44:313-329

9. Bowden J, Davey Smith G, Burgess S. Mendelian randomization with invalid instruments: effect estimation and bias detection through Egger regression. Int J Epidemiol 2015;44:512-525

10. Del Greco M F, Minelli C, Sheehan NA, Thompson JR. Detecting pleiotropy in Mendelian randomisation studies with summary data and a continuous outcome. Stat Med 2015;34:2926-2940

11. Staley JR, Burgess S. Semiparametric methods for estimation of a nonlinear exposure-outcome relationship using instrumental variables with application to Mendelian randomization. Genet Epidemiol 2017;41:341-352

12. Sun YQ, Burgess S, Staley JR, Wood AM, Bell S, Kaptoge SK, Guo Q, Bolton TR, Mason AM, Butterworth AS, Di Angelantonio E, Vie GA, Bjorngaard JH, Kinge JM, Chen Y, Mai XM. Body mass index and all cause mortality in HUNT and UK Biobank studies: linear and non-linear mendelian randomisation analyses. BMJ 2019;364:l1042

13. Burgess S, Davies NM, Thompson SG, Consortium EP-I. Instrumental variable analysis with a nonlinear exposure-outcome relationship. Epidemiology 2014;25:877-885

14. Didelez V, Sheehan N. Mendelian randomization as an instrumental variable approach to causal inference. Stat Methods Med Res 2007;16:309-330

15. Dupuis J, Langenberg C, Prokopenko I, Saxena R, Soranzo N, Jackson AU, Wheeler E, Glazer NL, Bouatia-Naji N, Gloyn AL, Lindgren CM, Magi R, Morris AP, Randall J, Johnson T, Elliott P, Rybin D, Thorleifsson G, Steinthorsdottir V, Henneman P, Grallert H,

Dehghan A, Hottenga JJ, Franklin CS, Navarro P, Song K, Goel A, Perry JRB, Egan JM, Lajunen T, Grarup N, Sparso T, Doney A, Voight BF, Stringham HM, Li M, Kanoni S, Shrader P, Cavalcanti-Proenca C, Kumari M, Qi L, Timpson NJ, Gieger C, Zabena C, Rocheleau G, Ingelsson E, An P, O'Connell J, Luan J, Elliott A, McCarroll SA, Payne F, Roccasecca RM, Pattou F, Sethupathy P, Ardlie K, Ariyurek Y, Balkau B, Barter P, Beilby JP, Ben-Shlomo Y, Benediktsson R, Bennett AJ, Bergmann S, Bochud M, Boerwinkle E, Bonnefond A, Bonnycastle LL, Borch-Johnsen K, Bottcher Y, Brunner E, Bumpstead SJ, Charpentier G, Chen YDI, Chines P, Clarke R, Coin LJM, Cooper MN, Cornelis M, Crawford G, Crisponi L, Day INM, de Geus EJC, Delplanque J, Dina C, Erdos MR, Fedson AC, Fischer-Rosinsky A, Forouhi NG, Fox CS, Frants R, Franzosi MG, Galan P, Goodarzi MO, Graessler J, Groves CJ, Grundy S, Gwilliam R, Gyllensten U, Hadjadj S, Hallmans G, Hammond N, Han XJ, Hartikainen AL, Hassanali N, Hayward C, Heath SC, Hercberg S, Herder C, Hicks AA, Hillman DR, Hingorani AD, Hofman A, Hui J, Hung J, Isomaa B, Johnson PRV, Jorgensen T, Jula A, Kaakinen M, Kaprio J, Kesaniemi YA, Kivimaki M, Knight B, Koskinen S, Kovacs P, Kyvik KO, Lathrop GM, Lawlor DA, Le Bacquer O, Lecoeur C, Li Y, Lyssenko V, Mahley R, Mangino M, Manning AK, Martinez-Larrad MT, McAteer JB, McCulloch LJ, McPherson R, Meisinger C, Melzer D, Meyre D, Mitchell BD, Morken MA, Mukherjee S, Naitza S, Narisu N, Neville MJ, Oostra BA, Orru M, Pakyz R, Palmer CNA, Paolisso G, Pattaro C, Pearson D, Peden JF, Pedersen NL, Perola M, Pfeiffer AFH, Pichler I, Polasek O, Posthuma D, Potter SC, Pouta A, Province MA, Psaty BM, Rathmann W, Rayner NW, Rice K, Ripatti S, Rivadeneira F, Roden M, Rolandsson O, Sandbaek A, Sandhu M, Sanna S, Sayer AA, Scheet P, Scott LJ, Seedorf U, Sharp SJ, Shields B, Sigurosson G, Sijbrands EJG, Silveira A, Simpson L, Singleton A, Smith NL, Sovio U, Swift A, Syddall H, Syvanen AC, Tanaka T, Thorand B, Tichet J, Tonjes A, Tuomi T, Uitterlinden AG, van Dijk KW, van Hoek M, Varma D, Visvikis-Siest S, Vitart V,

Vogelzangs N, Waeber G, Wagner PJ, Walley A, Walters GB, Ward KL, Watkins H, Weedon MN, Wild SH, Willemsen G, Witteman JCM, Yarnell JWG, Zeggini E, Zelenika D, Zethelius B, Zhai GJ, Zhao JH, Zillikens MC, Borecki IB, Loos RJF, Meneton P, Magnusson PKE, Nathan DM, Williams GH, Hattersley AT, Silander K, Salomaa V, Smith GD, Bornstein SR, Schwarz P, Spranger J, Karpe F, Shuldiner AR, Cooper C, Dedoussis GV, Serrano-Rios M, Morris AD, Lind L, Palmer LJ, Hu FB, Franks PW, Ebrahim S, Marmot M, Kao WHL, Pankow JS, Sampson MJ, Kuusisto J, Laakso M, Hansen T, Pedersen O, Pramstaller PP, Wichmann HE, Illig T, Rudan I, Wright AF, Stumvoll M, Campbell H, Wilson JF, Bergman RN, Buchanan TA, Collins FS, Mohlke KL, Tuomilehto J, Valle TT, Altshuler D, Rotter JI, Siscovick DS, Penninx BWJH, Boomsma DI, Deloukas P, Spector TD, Frayling TM, Ferrucci L, Kong A, Thorsteinsdottir U, Stefansson K, van Duijn CM, Aulchenko YS, Cao A, Scuteri A, Schlessinger D, Uda M, Ruokonen A, Jarvelin MR, Waterworth DM, Vollenweider P, Peltonen L, Mooser V, Abecasis GR, Wareham NJ, Sladek R, Froguel P, Watanabe RM, Meigs JB, Groop L, Boehnke M, McCarthy MI, Florez JC, Barroso I, Consortium D, Consortium G, Consortium GB, Consortiu AHP, Investigators M. New genetic loci implicated in fasting glucose homeostasis and their impact on type 2 diabetes risk. Nat Genet 2010;42:105-U132

16. Saxena R, Hivert MF, Langenberg C, Tanaka T, Pankow JS, Vollenweider P, Lyssenko V, Bouatia-Naji N, Dupuis J, Jackson AU, Kao WH, Li M, Glazer NL, Manning AK, Luan J, Stringham HM, Prokopenko I, Johnson T, Grarup N, Boesgaard TW, Lecoeur C, Shrader P, O'Connell J, Ingelsson E, Couper DJ, Rice K, Song K, Andreasen CH, Dina C, Kottgen A, Le Bacquer O, Pattou F, Taneera J, Steinthorsdottir V, Rybin D, Ardlie K, Sampson M, Qi L, van Hoek M, Weedon MN, Aulchenko YS, Voight BF, Grallert H, Balkau B, Bergman RN, Bielinski SJ, Bonnefond A, Bonnycastle LL, Borch-Johnsen K, Bottcher Y, Brunner E, Buchanan TA, Bumpstead SJ, Cavalcanti-Proenca C, Charpentier G, Chen YD, Chines PS,

Collins FS, Cornelis M, G JC, Delplanque J, Doney A, Egan JM, Erdos MR, Firmann M, Forouhi NG, Fox CS, Goodarzi MO, Graessler J, Hingorani A, Isomaa B, Jorgensen T, Kivimaki M, Kovacs P, Krohn K, Kumari M, Lauritzen T, Levy-Marchal C, Mayor V, McAteer JB, Meyre D, Mitchell BD, Mohlke KL, Morken MA, Narisu N, Palmer CN, Pakyz R, Pascoe L, Payne F, Pearson D, Rathmann W, Sandbaek A, Sayer AA, Scott LJ, Sharp SJ, Sijbrands E, Singleton A, Siscovick DS, Smith NL, Sparso T, Swift AJ, Syddall H, Thorleifsson G, Tonjes A, Tuomi T, Tuomilehto J, Valle TT, Waeber G, Walley A, Waterworth DM, Zeggini E, Zhao JH, consortium G, investigators M, Illig T, Wichmann HE, Wilson JF, van Duijn C, Hu FB, Morris AD, Frayling TM, Hattersley AT, Thorsteinsdottir U, Stefansson K, Nilsson P, Syvanen AC, Shuldiner AR, Walker M, Bornstein SR, Schwarz P, Williams GH, Nathan DM, Kuusisto J, Laakso M, Cooper C, Marmot M, Ferrucci L, Mooser V, Stumvoll M, Loos RJ, Altshuler D, Psaty BM, Rotter JI, Boerwinkle E, Hansen T, Pedersen O, Florez JC, McCarthy MI, Boehnke M, Barroso I, Sladek R, Froguel P, Meigs JB, Groop L, Wareham NJ, Watanabe RM. Genetic variation in GIPR influences the glucose and insulin responses to an oral glucose challenge. Nat Genet 2010;42:142-148

17. Willer CJ, Schmidt EM, Sengupta S, Peloso GM, Gustafsson S, Kanoni S, Ganna A, Chen J, Buchkovich ML, Mora S, Beckmann JS, Bragg-Gresham JL, Chang HY, Demirkan A, Den Hertog HM, Do R, Donnelly LA, Ehret GB, Esko T, Feitosa MF, Ferreira T, Fischer K, Fontanillas P, Fraser RM, Freitag DF, Gurdasani D, Heikkila K, Hypponen E, Isaacs A, Jackson AU, Johansson A, Johnson T, Kaakinen M, Kettunen J, Kleber ME, Li X, Luan J, Lyytikainen LP, Magnusson PKE, Mangino M, Mihailov E, Montasser ME, Muller-Nurasyid M, Nolte IM, O'Connell JR, Palmer CD, Perola M, Petersen AK, Sanna S, Saxena R, Service SK, Shah S, Shungin D, Sidore C, Song C, Strawbridge RJ, Surakka I, Tanaka T, Teslovich TM, Thorleifsson G, Van den Herik EG, Voight BF, Volcik KA, Waite LL, Wong A, Wu Y, Zhang W, Absher D, Asiki G, Barroso I, Been LF, Bolton JL, Bonnycastle LL, Brambilla P,

Burnett MS, Cesana G, Dimitriou M, Doney ASF, Doring A, Elliott P, Epstein SE, Ingi Eyjolfsson G, Gigante B, Goodarzi MO, Grallert H, Gravito ML, Groves CJ, Hallmans G, Hartikainen AL, Hayward C, Hernandez D, Hicks AA, Holm H, Hung YJ, Illig T, Jones MR, Kaleebu P, Kastelein JJP, Khaw KT, Kim E, Klopp N, Komulainen P, Kumari M, Langenberg C, Lehtimaki T, Lin SY, Lindstrom J, Loos RJF, Mach F, McArdle WL, Meisinger C, Mitchell BD, Muller G, Nagaraja R, Narisu N, Nieminen TVM, Nsubuga RN, Olafsson I, Ong KK, Palotie A, Papamarkou T, Pomilla C, Pouta A, Rader DJ, Reilly MP, Ridker PM, Rivadeneira F, Rudan I, Ruokonen A, Samani N, Scharnagl H, Seeley J, Silander K, Stancakova A, Stirrups K, Swift AJ, Tiret L, Uitterlinden AG, van Pelt LJ, Vedantam S, Wainwright N, Wijmenga C, Wild SH, Willemsen G, Wilsgaard T, Wilson JF, Young EH, Zhao JH, Adair LS, Arveiler D, Assimes TL, Bandinelli S, Bennett F, Bochud M, Boehm BO, Boomsma DI, Borecki IB, Bornstein SR, Bovet P, Burnier M, Campbell H, Chakravarti A, Chambers JC, Chen YI, Collins FS, Cooper RS, Danesh J, Dedoussis G, de Faire U, Feranil AB, Ferrieres J, Ferrucci L, Freimer NB, Gieger C, Groop LC, Gudnason V, Gyllensten U, Hamsten A, Harris TB, Hingorani A, Hirschhorn JN, Hofman A, Hovingh GK, Hsiung CA, Humphries SE, Hunt SC, Hveem K, Iribarren C, Jarvelin MR, Jula A, Kahonen M, Kaprio J, Kesaniemi A, Kivimaki M, Kooner JS, Koudstaal PJ, Krauss RM, Kuh D, Kuusisto J, Kyvik KO, Laakso M, Lakka TA, Lind L, Lindgren CM, Martin NG, Marz W, McCarthy MI, McKenzie CA, Meneton P, Metspalu A, Moilanen L, Morris AD, Munroe PB, Njolstad I, Pedersen NL, Power C, Pramstaller PP, Price JF, Psaty BM, Quertermous T, Rauramaa R, Saleheen D, Salomaa V, Sanghera DK, Saramies J, Schwarz PEH, Sheu WH, Shuldiner AR, Siegbahn A, Spector TD, Stefansson K, Strachan DP, Tayo BO, Tremoli E, Tuomilehto J, Uusitupa M, van Duijn CM, Vollenweider P, Wallentin L, Wareham NJ, Whitfield JB, Wolffenbuttel BHR, Ordovas JM, Boerwinkle E, Palmer CNA, Thorsteinsdottir U, Chasman DI, Rotter JI, Franks PW, Ripatti S, Cupples LA, Sandhu MS, Rich SS, Boehnke

M, Deloukas P, Kathiresan S, Mohlke KL, Ingelsson E, Abecasis GR, Global Lipids Genetics C. Discovery and refinement of loci associated with lipid levels. Nat Genet 2013;45:1274-1283

ESM Table 1. The genetic variants used as instruments for average blood glucose levels, and their associations with type 2 diabetes liability and HbA_{1c}. Genetic variants were selected based on their association with type 2 diabetes liability ($p<5x10^{-8}$) in a genome-wide association study of 228,499 cases and 1,178,783 controls (79% European ancestry) that included UK Biobank participants and their association with HbA_{1c} ($p<0.001$ and concordant direction of association) in an independent study of 100,880 European ancestry participants (no overlap with UK Biobank) that were free of diabetes mellitus (as defined by physician diagnosis, medications, or fasting glucose ≥7 mmol/L).

						Type 2 diabetes			HbA_{1c}		
Single-nucleotide polymorphism	Chromosome	Position	Effect allele	Other allele	Effect allele frequency	Beta (log odds ratio)	Standard error	*p* value	Beta (%)	Standard error	*p* value
rs10923360	1	118166877	T	C	0.3299	0.0286	0.004	5.99E-13	0.007	0.0019	1.73E-04
rs3020781	1	155269776	G	A	0.3719	0.0294	0.0043	7.29E-12	0.0069	0.002	4.53E-04
rs340874	1	214159256	C	T	0.4991	0.0543	0.0039	6.47E-45	0.0079	0.0016	3.65E-07
rs1260326	2	27730940	C	T	0.5805	0.0625	0.0039	2.16E-57	0.0059	0.0017	3.69E-04
rs17334919	2	43707385	T	C	0.0916	-0.1207	0.0075	2.27E-58	-0.012	0.003	6.44E-05
rs10184004	2	165508389	T	C	0.4049	-0.0636	0.0041	4.39E-54	-0.0063	0.0018	3.45E-04
rs11708067	3	123065778	G	A	0.2229	-0.0804	0.005	1.63E-57	-0.013	0.0019	1.42E-12
rs9873519	3	124921457	T	C	0.4712	0.0373	0.0038	2.53E-22	0.0071	0.0019	1.39E-04
rs16851397	3	141134818	G	A	0.0656	-0.0684	0.0093	2.17E-13	-0.014	0.0037	1.25E-04
rs8192675	3	170724883	C	T	0.3167	-0.0452	0.0042	3.85E-27	-0.011	0.0017	1.38E-11
rs9859406	3	185534482	A	G	0.3524	0.1117	0.004	2.01E-169	0.0064	0.0019	5.95E-04
rs1996617	4	52798624	C	T	0.3914	0.0289	0.004	6.54E-13	0.0078	0.002	7.35E-05
rs735949	4	185716232	C	T	0.1332	-0.0555	0.0064	2.78E-18	-0.0098	0.0022	6.58E-06
rs6878122	5	76427311	A	G	0.7058	-0.0517	0.0045	2.00E-30	-0.0091	0.0019	1.05E-06
rs10440833	6	20688121	T	A	0.7028	-0.1286	0.0041	4.51E-215	-0.01	0.002	1.65E-07
rs3117189	6	32033944	G	A	0.8469	0.0573	0.0057	3.87E-24	0.013	0.0031	1.61E-05

rs679582	6	139831180	A	G	0.5485	-0.0262	0.0039	2.28E-11	-0.009	0.0019	1.37E-06
rs2191349	7	15064309	T	G	0.5668	0.067	0.0038	2.57E-71	0.0086	0.0017	2.09E-07
rs2267716	7	30716643	C	T	0.2741	-0.0364	0.0044	2.21E-16	-0.0074	0.0022	6.66E-04
rs1799884	7	44229068	T	C	0.1716	0.0553	0.005	9.45E-29	0.029	0.0026	1.06E-29
rs13266634	8	118184783	T	C	0.3186	-0.1024	0.0041	4.23E-136	-0.015	0.0017	4.53E-20
rs4237150	9	4290085	C	G	0.4292	0.0433	0.0037	6.98E-31	0.0064	0.0018	2.77E-04
rs10811661	9	22134094	C	T	0.2442	-0.1471	0.0048	9.59E-206	-0.014	0.0024	4.14E-09
rs505922	9	136149229	C	T	0.3613	0.0413	0.0039	1.61E-26	0.0072	0.0017	1.39E-05
rs11257655	10	12307894	T	C	0.2956	0.0946	0.0056	1.44E-63	0.0082	0.0021	7.51E-05
rs1111875	10	94462882	T	C	0.451	-0.0928	0.0038	1.44E-128	-0.0068	0.0016	1.20E-05
rs17747324	10	114752503	C	T	0.2198	0.2493	0.005	<1.00E-299	0.015	0.0023	6.12E-11
rs2403221	11	9852475	A	G	0.5516	0.0263	0.0041	1.16E-10	0.0065	0.0019	4.88E-04
rs757110	11	17418477	A	C	0.6296	-0.0599	0.0039	4.59E-52	-0.0056	0.0017	7.25E-04
rs174541	11	61565908	C	T	0.3493	-0.0277	0.0041	2.39E-11	-0.0076	0.002	1.12E-04
rs1552224	11	72433098	C	A	0.1452	-0.0917	0.0058	2.53E-56	-0.012	0.0021	1.61E-08
rs10830963	11	92708710	G	C	0.3057	0.0731	0.0042	1.29E-66	0.02	0.002	2.23E-23
rs2732480	12	48736303	A	C	0.4201	-0.0314	0.0042	6.40E-14	-0.012	0.002	2.00E-09
rs12910361	15	77782335	G	A	0.614	0.0686	0.004	5.19E-65	0.008	0.002	4.78E-05
rs2290202	15	91512267	T	G	0.2533	0.0576	0.005	6.31E-31	0.011	0.0029	1.29E-04
rs6600191	16	295795	C	T	0.2491	-0.0428	0.0046	2.97E-20	-0.01	0.0026	5.88E-05
rs1421085	16	53800954	C	T	0.367	0.1182	0.004	1.29E-189	0.0085	0.0017	2.89E-07
rs2297508	17	17715317	G	C	0.5583	-0.0305	0.004	2.39E-14	-0.0068	0.0019	2.64E-04
rs12603589	17	65825248	C	T	0.3128	0.0421	0.0046	1.06E-19	0.0075	0.0023	9.94E-04
rs10408179	19	46157004	C	T	0.4257	-0.0517	0.0039	1.86E-40	-0.006	0.0017	2.93E-04

ESM Table 2. Baseline characteristics of UK Biobank participants. Individuals with possible diabetes mellitus were excluded. CHD: coronary heart disease; LDL-C: low-density lipoprotein cholesterol; SD: standard deviation.

	**Overall**	**Males**	**Females**	**No CHD event**	**CHD event**	**Possible diabetes mellitus (excluded)**
**Number of participants (n)**	324,830	145,472	179,358	318,824	6006	26,562
**Mean (SD) age at survey / years**	56.9 (8.0)	57.1 (8.2)	56.8 (7.9)	56.9 (8.1)	60.5 (6.9)	60.1 (7.1)
**Number (%) of females**	179,358 (55.2)	-	-	177,758 (55.8)	1600 (26.6)	10,493 (39.5)
**Mean (SD) body mass index / kg/m^2**	27.0 (4.5)	27.5 (3.9)	26.7 (4.9)	27.0 (4.5)	28.0 (4.3)	31.6 (5.8)
**Mean (SD) HbA$_{1c}$ / mmol/mol**	34.8 (3.6)	34.8 (3.9)	34.9 (3.6)	34.8 (3.6)	35.9 (3.7)	49.9 (14.0)
**Mean (SD) HbA$_{1c}$ / %**	5.3 (0.3)	5.3 (0.3)	5.3 (0.3)	5.3 (0.3)	5.4 (0.3)	6.7 (1.3)
**Mean (SD) LDL-cholesterol / mmol/L**	3.6 (0.8)	3.6 (0.8)	3.7 (0.9)	3.6 (0.8)	3.7 (1.0)	3.0 (0.9)
**Mean (SD) systolic blood pressure / mmHg**	137.3 (18.6)	140.6 (17.4)	134.7 (19.2)	137.2 (18.6)	144.8 (19.6)	142.1 (18.0)
**Mean (SD) diastolic blood pressure / mmHg**	81.9 (10.1)	84.0 (10.0)	80.3 (9.9)	81.9 (10.1)	84.5 (10.7)	82.3 (10.3)
**Number of current smokers (%)**	33,052 (10.2)	17,566 (12.1)	15,486 (8.6)	31,834 (10.0)	1218 (20.3)	3213 (12.1)
**Number of current alcohol drinkers (%)**	304,235 (93.7)	138,544 (95.2)	165,691 (92.4)	298,726 (93.7)	5509 (91.7)	23,208 (87.4)

ESM Table 3. Results of non-linear Mendelian randomization analyses investigating the association of genetically-proxied average blood glucose levels with incident coronary heart disease in the total analytic sample (all) and in quintiles of the sample based on residual HbA_{1c}. Hazard ratios are given per 1 mmol/mol increase in genetically-proxied HbA_{1c}. HR: hazard ratio.

Strata	Overall		Males		Females	
	**Mean HbA_{1c} (mmol/mol / %)**	**HR (95% CI)**	**Mean HbA_{1c} (mmol/mol / %)**	**HR (95% CI)**	**Mean HbA_{1c} (mmol/mol / %)**	**HR (95% CI)**
All	34.8 / 5.3%	1.11 (1.05-1.18) $p=2\times10^{-4}$	34.8 / 5.3%	1.12 (1.05-1.19) $p=4\times10^{-4}$	34.9 / 5.3%	1.08 (0.96-1.20) $p=0.20$
Quintile 1	29.8 / 4.9%	1.18 (1.01-1.37) $p=0.037$	29.8 / 4.9%	1.22 (1.03-1.45) $p=0.023$	29.9 / 4.9%	1.07 (0.78-1.48) $p=0.67$
Quintile 2	33.0 / 5.2%	1.10 (0.95-1.26) $p=0.21$	33.0 / 5.2%	1.13 (0.96-1.32) $p=0.13$	33.1 / 5.2%	1.05 (0.77-1.42) $p=0.77$
Quintile 3	34.8 / 5.3%	1.27 (1.11-1.45) $p=4\times10^{-4}$	34.8 / 5.3%	1.22 (1.05-1.41) $p=0.010$	34.9 / 5.3%	1.28 (0.98-1.66) $p=0.066$
Quintile 4	36.6 / 5.5%	1.10 (0.98-1.24) $p=0.12$	36.6 / 5.5%	1.08 (0.95-1.24) $p=0.24$	36.7 / 5.5%	1.15 (0.91-1.45) $p=0.26$
Quintile 5	39.8 / 5.8%	1.01 (0.91-1.12) $p=0.90$	39.8 / 5.8%	1.04 (0.92-1.17) $p=0.53$	39.8 / 5.8%	0.93 (0.76-1.13) $p=0.46$

ESM Table 4. Results of linear Mendelian randomization (MR) sensitivity analyses investigating the association of genetically-proxied average blood glucose levels with incident coronary heart disease. Hazard ratios are given per 1mmol/mol increase in genetically-proxied HbA_{1c}. HR: hazard ratio.

**Method**	**Hazard ratio (95% CI)**	***p* value**
**Inverse-variance weighted method (fixed-effects)**	1.11 (1.05, 1.18)	$2×10^{-4}$
**Inverse-variance weighted method (random-effects)**	1.11 (1.03, 1.21)	0.007
**Weighted median method**	1.12 (1.03, 1.23)	0.009
**MR-Egger method**	1.11 (0.91, 1.35)	0.30
**(intercept)**	0.000 (-0.022, 0.022)	0.97
**MR-PRESSO method**	1.10 (1.02, 1.18)	0.014
**Contamination mixture method**	1.15 (1.06, 1.24)	$4×10^{-4}$
**Heterogeneity test**	**Statistic**	***p* value**
**Q statistic**	73.1	$8×10^{-4}$
**I^2 statistic**	46.7%	

One variant was excluded from analysis in the MR-PRESSO method (rs505922).

ESM Table 5. Results of Mendelian randomization (MR) analyses investigating the association of genetically-proxied average blood glucose levels with glycaemic and lipid traits. Estimates are given per 1mmol/mol increase in genetically-proxied HbA_{1c}.

Trait	Estimate (95% CI)	*p* value
**Fasting glucose (mmol/L)**	1.72 (1.36, 2.07)	<0.001
**Two-hour glucose (mmol/L)**	3.13 (1.95, 4.32)	<0.001
**Fasting insulin (nmol/L, log-transformed)**	-0.133 (-0.347, 0.080)	0.22
**Low-density lipoprotein-cholesterol (SD units)**	0.433 (0.034, 0.831)	0.033
**High-density lipoprotein-cholesterol (SD units)**	-0.031 (-0.378, 0.316)	0.86
**Triglycerides (SD units)**	0.000 (-0.794, 0.794)	0.99

ESM Figure 1. Genetic associations of the instrument variants with HbA_{1c} (mmol/mol units) in male and female participants of the UK Biobank respectively, obtained from analyses performed by the Neale Lab (available at http://www.nealelab.is/uk-biobank). Association estimates for the rs2732480 variant were missing. Error bars represent 95% confidence intervals.

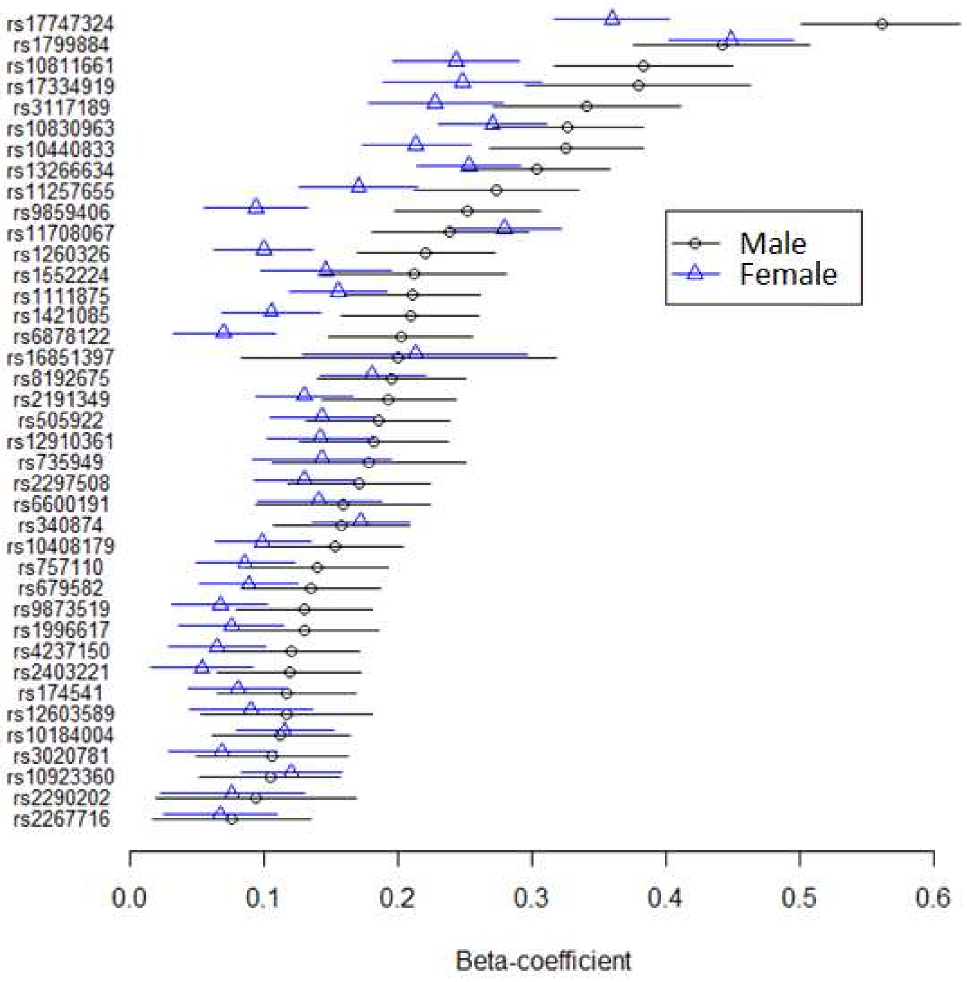

ESM Figure 2. Scatter plot of genetic association estimates for HbA_{1c} and incident coronary heart disease (CHD) risk. The gradient of the blue line depicts the random effects inverse-variance weighted Mendelian randomization estimate. For each variant (N=40), the genetic association and its 95% confidence interval with the exposure (HbA_{1c}; x-axis) and with the outcome (CHD risk; y-axis, log odds ratio) are plotted.

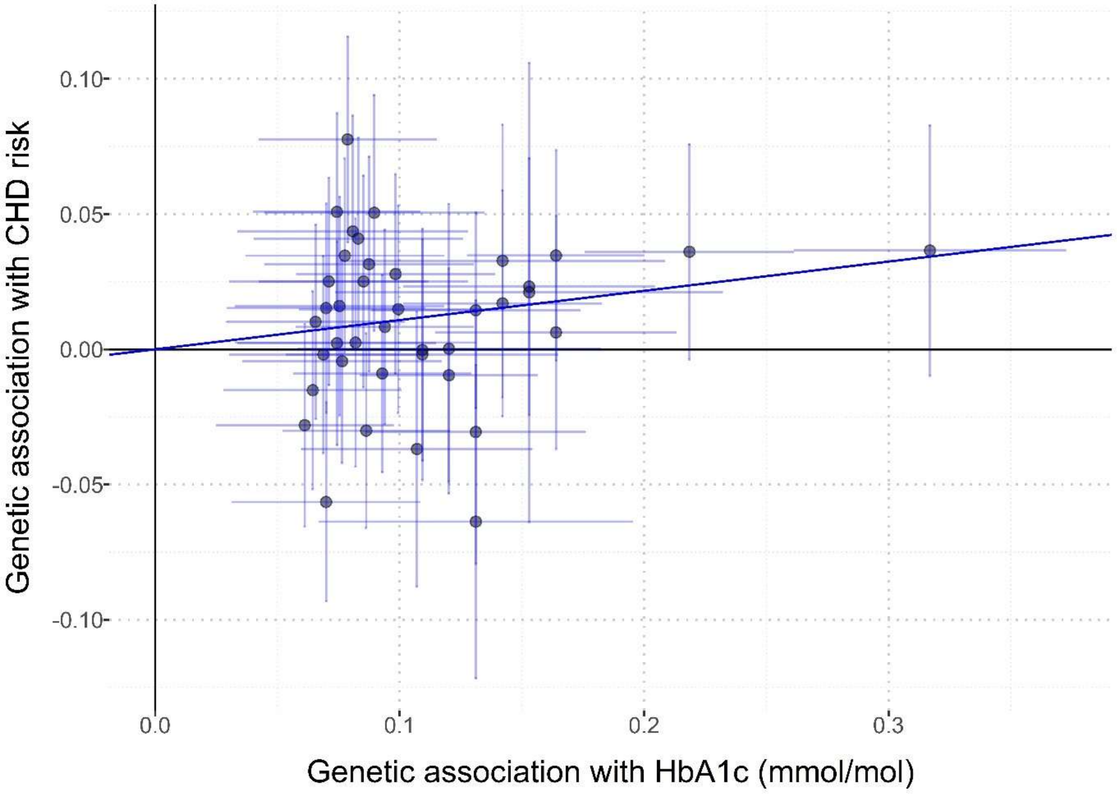

ESM Figure 3. Scatter plots of genetic association estimates for HbA_{1c} and glycaemic and lipid traits, for each variant (N=40).

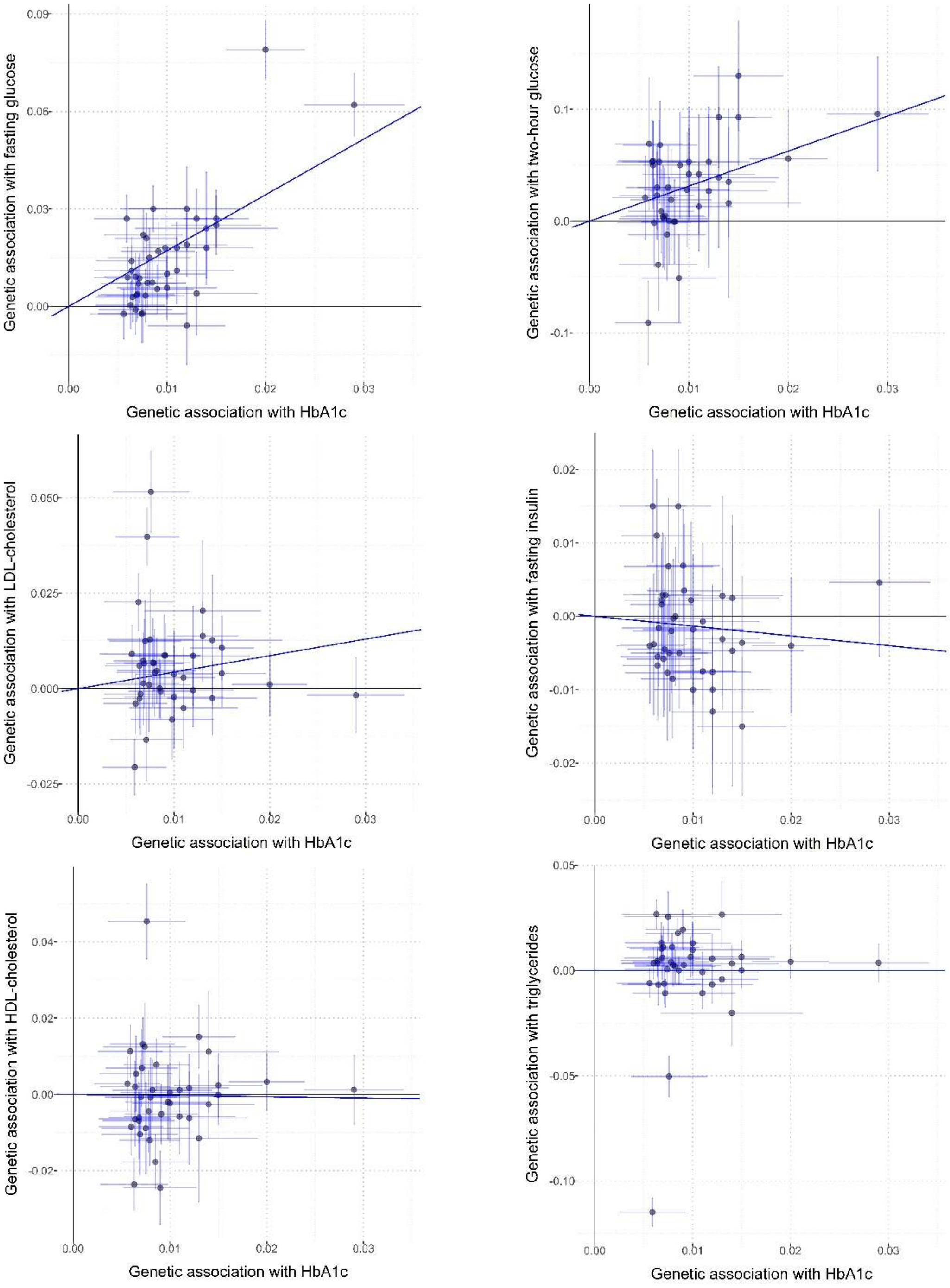

ESM Figure 4. Multivariable Mendelian randomization (adjusting for genetically-proxied low-density lipoprotein cholesterol) investigating the relationship between genetically-proxied average blood glucose levels, as measured by HbA_{1c}, and risk of incident coronary heart disease in individuals without diabetes mellitus in males and females combined. The x-axis depicts HbA_{1c} levels in mmol/mol. The y-axis depicts the hazard ratio for coronary heart disease with respect to the reference. Reference is set to an HbA_{1c} of 30mmol/mol (4.9%). The grey lines represent the 95% confidence intervals. The fractional polynomial test is a goodness-of-fit test that assesses whether any improvement of fit when using a non-linear function to model the association, as compared to a linear function, is greater than would be expected due to chance (a significant *p* value indicates that a non-linear model is preferred to a linear model).

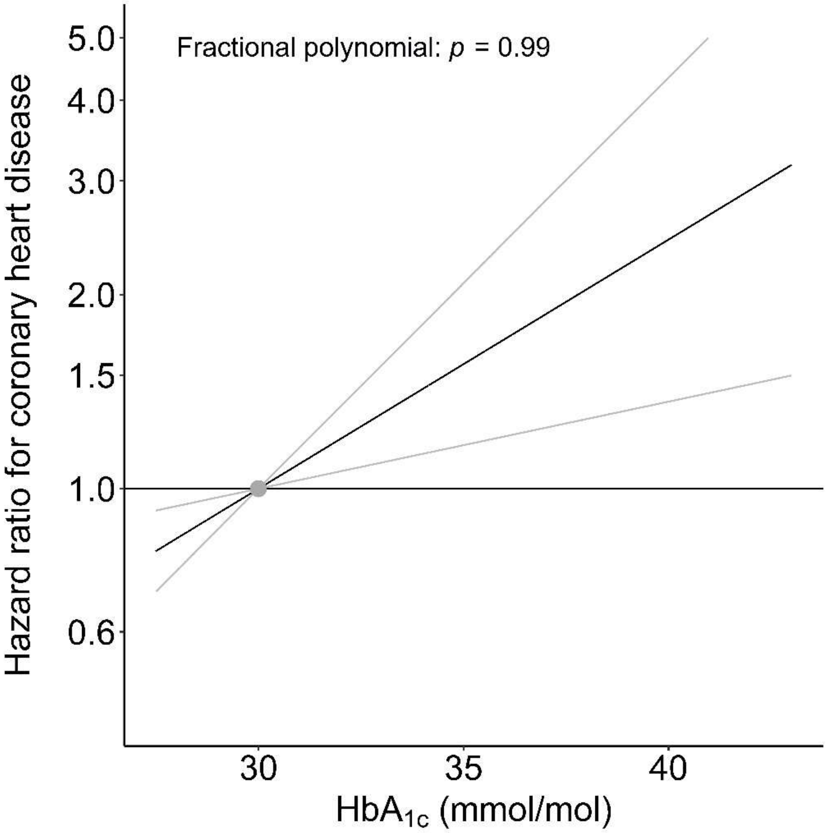

ESM Figure 5. Non-linear Mendelian randomization investigating the relationship between genetically-proxied average blood glucose levels, as measured by HbA_{1c}, and risk of incident coronary heart disease in individuals without diabetes mellitus in males and females combined. The five variants that associated with low-density lipoprotein cholesterol at $p<0.01$ (rs1260326, rs10184004, rs11708067, rs505922 and rs174541) were excluded. Three of these variants (s10184004, rs505922 and rs174541) associated with low-density lipoprotein cholesterol at $p<5\text{x}10^{-8}$). The x-axis depicts HbA_{1c} levels in mmol/mol. The y-axis depicts the hazard ratio for coronary heart disease with respect to the reference. Reference is set to an HbA_{1c} of 30mmol/mol (4.9%). The grey lines represent the 95% confidence intervals. The fractional polynomial test is a goodness-of-fit test that assesses whether any improvement of fit when using a non-linear function to model the association, as compared to a linear function, is greater than would be expected due to chance (a significant p value indicates that a non-linear model is preferred to a linear model).

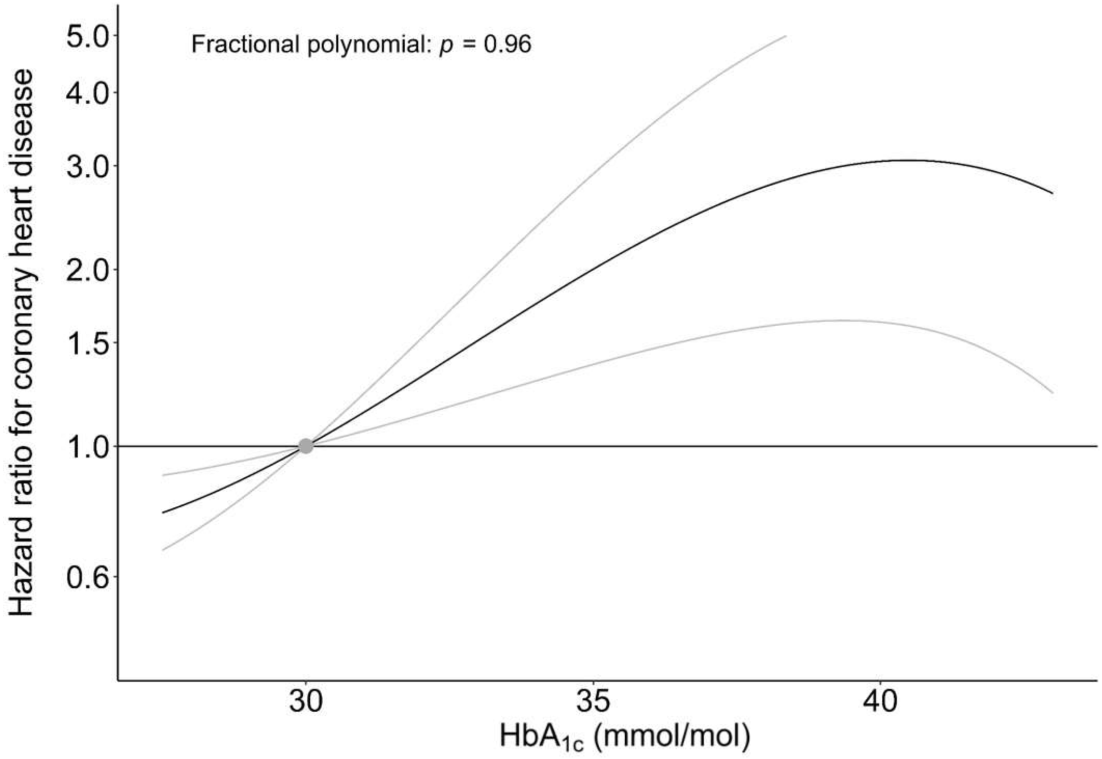

Supplement: Supplementary file 1 — (PDF 980 kb) [file 125_2020_5377_MOESM1_ESM.pdf]
